# Photothermally Controlled Methotrexate Release System Using β-Cyclodextrin and Gold Nanoparticles

**DOI:** 10.3390/nano8120985

**Published:** 2018-11-28

**Authors:** Nataly Silva, Ana Riveros, Nicolás Yutronic, Erika Lang, Boris Chornik, Simón Guerrero, Josep Samitier, Paul Jara, Marcelo J. Kogan

**Affiliations:** 1 Department of Chemistry, University of Chile, Las Palmeras 3425, 7800003 Santiago, Chile; n.silvag@ug.uchile.cl (N.S.); nyutroni@uchile.cl (N.Y.); 2 Department of Chemistry of Materials, University of Santiago de Chile, Av. Libertador Bernardo O’Higgins 3363, 9170022 Santiago, Chile; 3 Department of Pharmacological and Toxicological Chemistry, University of Chile, Sergio Livingston 1007, 8380492 Santiago, Chile; ariveros@postqyf.uchile.cl (A.R.); simon.daiblogt@gmail.com (S.G.); 4 Advanced Center for Chronic Diseases (ACCDiS), University of Chile, Sergio Livingstone 1007, 8380000 Independencia Santiago, Chile; 5 Department of Biology, CEM, University of Chile, Las Palmeras 3425, 7800003 Santiago, Chile; Elang@uchile.cl; 6 Department of Physics, University of Chile, Beauchef 850, 8370448 Santiago, Chile; bchornik@ing.uchile.cl; 7 Nanobioengineering Laboratory, Institute for Bioengineering of Catalonia (IBEC), Barcelona Institute of Science and Technology (BIST) Baldiri Reixac, 10–12, 08028 Barcelona, Spain; jsamitier@ibecbarcelona.eu; 8 Centro de Investigación Biomédica en Red en Bioingeniería, Biomateriales y Nanomedicina (CIBER-BBN), Monforte de Lemos 3–5, Pabellón 11, 28029 Madrid, Spain; 9 Department of Electronics and Biomedical Engineering, University of Barcelona (UB), Martí I Franques, 1, 08028 Barcelona, Spain

**Keywords:** inclusion compound, cyclodextrin, Methotrexate, delivery system, gold nanoparticles, photothermal release, laser, irradiation

## Abstract

The inclusion compound (IC) of cyclodextrin (CD) containing the antitumor drug Methotrexate (MTX) as a guest molecule was obtained to increase the solubility of MTX and decrease its inherent toxic effects in nonspecific cells. The IC was conjugated with gold nanoparticles (AuNPs), obtained by a chemical method, creating a ternary intelligent delivery system for MTX molecules, based on the plasmonic properties of the AuNPs. Irradiation of the ternary system, with a laser wavelength tunable with the corresponding surface plasmon of AuNPs, causes local energy dissipation, producing the controlled release of the guest from CD cavities. Finally, cell viability was evaluated using MTS assays for β-CD/MTX and AuNPs + β-CD/MTX samples, with and without irradiation, against HeLa tumor cells. The irradiated sample of the ternary system AuNPs + β-CD/MTX produced a diminution in cell viability attributed to the photothermal release of MTX.

## 1. Introduction

Studies on the usage of nanoparticles (NPs) in the field of medicine have increased considerably in recent years mainly because NPs can act as vectors for the diagnosis and treatment of several diseases. These studies highlight the role of gold nanoparticles (AuNPs) in drug delivery, targeting and imaging applications as alternative therapies for cancer. AuNPs improve drug delivery to tumors through passive and active vectorization [1,2]. Passive vectorization involves the accumulation of NPs in tumor tissue due to their enhanced permeability and retention through the pores of the blood vessels, whereas active vectorization involves conjugation to the NPs of molecules that are capable of specifically recognizing the therapeutic target. A nanocarrier based on NPs, which combines both the vectorization mechanisms, is considered to be an ideal carrier. Furthermore, the optical properties of AuNPs allow external activation with an appropriate electromagnetic source [3,4,5]. This process is advantageous to perform cancer treatment using the following two possible mechanisms: (a) local hyperthermic effects of AuNPs and (b) release of a potential antitumor drug conjugated to AuNPs, via irradiation. Therefore, through the use of AuNPs, it is possible to improve the traditional cancer treatments that exhibit severe limitations mainly related to the low specificity of the chemotherapeutic agents to the tumor cells. AuNPs are useful as a drug delivery system that allows to spatially and temporally control the release of drugs in the tumor to avoid unspecific effects. AuNPs can be conjugated to antitumor drugs and after external localized stimuli with a laser, it is possible to trigger the release of the drugs in the affected tissue due to the local photothermal effect [6,7,8,9].

Methotrexate (MTX) is formed from a pteridine ring, a p-aminobenzoic acid residue, and a glutamic acid residue. Substitution of 4-hydroxyl with an amino group creates a folic acid analog, which is an antimetabolite. MTX competitively inhibits the enzyme of dihydrofolate reductase (DHFR) and impedes the formation of tetrahydrofolate, which is required to synthesize essential purines and pyrimidines, which are involved in both DNA and RNA synthesis. Therefore, MTX is considered to be an antifolate, which can be recognized by its pteridine ring by folate receptors that are overexpressed on the surfaces of various cells [10]. MTX was considered to be an effective antineoplastic to treat cell proliferation disorders such as lymphocytic leukemia; breast carcinoma; tongue, pharynx, bladder, and brain tumors; and skin neoplasms [10,11,12]. However, it also exhibits several undesirable side effects such as stomach irritation, nausea, and dizziness caused by the administration of high dosages of MTX, due to the tumor cells possibly being drug resistant, increasing the risk of toxicity [13]. Such effects are related to the poor selectivity in MTX distribution. Therefore, developing intelligent systems for drug delivery is essential. One strategy is to encapsulate the MTX within a matrix, such as CD, to form IC with the objective to increase the drug solubility and avoid interaction with the folate receptors in non-tumor cells [14,15,16]. Ensuring the release of MTX in the tumor will allow for a considerably high selectivity of action. Another strategy is to conjugate the MTX on the surface of AuNPs to obtain a nanotransporter that can be actively and passively vectorized [17,18,19,20,21].

In previous studies, we obtained ternary systems that comprised 1:2 α-cyclodextrin-octylamine (α-CD-OA) [22,23] and 1:1 β-cyclodextrin–phenylethylamine (β-CD-PhEA) [24]. The ICs were conjugated to AuNPs that were obtained by chemical or physical methods, respectively. We reported the effects of green-laser irradiation of AuNPs on the IC structure and demonstrated that the heat generated in the irradiated nanostructure was transferred to the supramolecular structure, which caused the disassembly of the AuNP-IC system that further caused the guest release from the CD cavity by topotactic transition.

**Figure 1:**
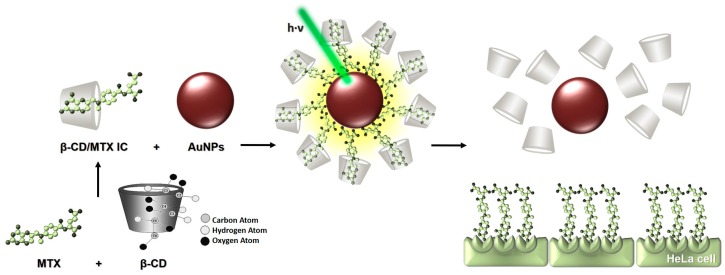
Schematic representation of the precursors MTX and β-CD to form the inclusion compound (IC) and subsequently of the ternary system, where the IC was conjugated to the AuNP. Laser light is absorbed by the AuNP and is transformed into local heat, which is dissipated into the environment and induces the release of MTX from the CD cavity. Further, the free drug interacts with the folate receptors in the HeLa cells.

In this study, we obtained and characterized a ternary system formed from MTX, β-CD, and AuNPs (Figure 1) that were obtained by Turkevich synthesis. We irradiated the complex and evaluated, for the first time, the biological activity in the HeLa cells; thus, we observed that the effects of MTX on cell viability are dependent on the irradiation of the ternary complex.

## 2. Experimental

### 2.1. Purification of MTX

A lyophilized vial of MTX (500 mg) with mannitol and NaOH was donated by the Kampar Oncology Laboratory (Santiago, Chile). Therefore, it was necessary to separate the drug from the excipients before usage. The lyophilized compounds were dissolved in nanopure water (Merck, Darmstadt, Germany) (20 mL). The solution was further acidified using 0.1 M HCl (Sigma-Aldrich, St. Louis, MO, USA) to precipitate the drug because it was insoluble at a pH value of 4. The solution was further centrifuged at 1485×*g* and was washed thrice using water, ensuring the complete elimination of mannitol. Proton nuclear magnetic resonance (^1^H-NMR) was used to verify the elimination of the excipients from the sample and to characterize the structure of MTX (characterization by ^1^H-NMR, see Figure S1 and Table S1). No signals corresponding to mannitol [25] were observed after the MTX purification.

### 2.2. Preparation and Characterization of the β-CD/MTX Inclusion Compound

Equimolar amounts of MTX crystals and β-CD (Sigma-Aldrich, Saint Louis, MO, USA) (1 g of β-CD and 0.4 g of MTX) were mixed in 10 mL water, with continuous and vigorous stirring, at room temperature for 48 h. The solution was left to crystallize for 7 days. The obtained solid was washed using water and dried at 60 °C (yields: β-CD/MTX 64.25%). The inclusion compound (IC) was characterized using NMR spectroscopy. All the spectra were recorded at 400 MHz and a temperature of 27 °C on a Bruker Avance 400 MHz superconducting NMR spectrometer in dimethyl sulfoxide-d_6_ (DMSO-d_6_) (Sigma-Aldrich, Saint Louis, MO, USA). To conduct the 2D-ROESY (Rotating-frame Overhauser Spectroscopy) NMR, the WG-ROESY (Watergate-ROESY) pulse sequence was used, and 2D-ROESY measurements were performed under the following experimental conditions: 64 scans; an acquisition time of 0.150 s; a pulse delay of 8 s; and 1024 data points.

### 2.3. Synthesis and Characterization of Colloidal AuNPs and Their Conjugation with the β-CD/MTX

The following chemical reagents were commercially available and were used as received: tetrachloroauric (III) acid (Sigma-Aldrich, Saint Louis, MO, USA); sodium citrate (Sigma-Aldrich, Saint Louis, MO, USA); sodium hydroxide (Sigma-Aldrich, Saint Louis, MO, USA); and sodium phosphate buffer (PBS) (Sigma-Aldrich, Saint Louis, MO, USA).

The AuNPs were synthesized in accordance with the Turkevich method [26]. In a round-bottom flask equipped with a condenser, 1.0 mM aqueous HAuCl_4_ (100 mL) was brought to rolling boil with vigorous stirring; further, 38.8 mM aqueous Na_3_C_6_H_5_O_7_ (10 mL) was rapidly added to the solution while stirring continuously. The solution was heated for an additional period of 30 min and was then maintained at room temperature. It was further filtered through a 0.45-μm cellulose acetate membrane filter. The β-CD/MTX IC was conjugated to the AuNPs using the same protocol as that used for the conjugation of MTX to AuNPs [17]. The AuNPs suspension was eight times concentrated in amicon ultra centrifugal filters (500 µL) and 200 mM IC (5 µL) was added to 10 mM PBS (500 µL). The solution was stirred for 24 h at room temperature (Schematic representation of the methodologic protocol, Figure S2).

Further, the solution was centrifuged at 13,362× *g* for 20 min, and the precipitate was separated from the supernatant and the pellet was resuspended in 10 mM PBS (1 mL).

The AuNPs and the conjugated AuNPs + MTX and AuNPs + β-CD/MTX were characterized by UV-Vis spectrophotometry. This confirmed the conjugation of both MTX and IC on the surface of AuNPs by displacing the characteristic surface plasmon band of the AuNPs. A Shimadzu UV-3101PC spectrophotometer (Kyoto, Japan) was used. The spectra were recorded between 280 and 800 nm.

The morphologies and sizes of the AuNPs and conjugates were determined by transmission electron microscopy (TEM). TEM was performed using a JEOL JEM-1010 transmission electron microscope (120 keV, Peabody, MA, USA). The sample was dropped on a copper mesh that was coated with carbon and Formvar.

The size was also determined by dynamic light scattering (DLS). The DLS spectra were obtained in triplicate on Malvern Instruments Zetasizer Nano S90 (Worcestershire, England, UK) using 1-cm plastic cuvettes.

X-ray photoelectron spectroscopy (XPS) was used to determine the energy that was associated with the interactions between the IC and AuNPs. The XPS spectra were recorded on a Perkin Elmer 1257 photoelectron spectrometer (Eden Prairie, MN, USA) fitted with an ultra-high vacuum main chamber (Eden Prairie, MN, USA), a hemispherical electron analyzer (Eden Prairie, MN, USA), and an X-ray source providing unfiltered Al Kα radiation. Energy calibration was performed by assigning a binding energy of 284.8 eV to the C-C component at the 1 s peak.

Electrophoresis was used to qualitatively determine the charges on the compounds by comparing the mobilities of various compounds in an electric field. Twenty-five-fold concentrated samples (AuNPs, AuNPs + MTX, and AuNPs + IC, 20 µL) were further mixed with 10 µL of the loading buffer (70% glycerol with 30% 1 × Tris-acetate–EDTA (TAE)). The samples were subjected to 1% agarose gel electrophoresis at 130 V for 10 min in 1 × TAE.

The AuNPs + β-CD/MTX sample was characterized by thermal analysis with the aim to determine the stability and thermal events, and to provide information about the physical properties of the samples. Thermogravimetric analysis (TGA) was performed using a thermobalance (TGA-SDTA 851e/SF/1100, Mettler Toledo, Greifensee, Switzerland). The sample was placed in an aluminum capsule. Nitrogen was used as the purge gas, and the temperature range was 25–200 °C, with a heating rate of 10 °C min^−1^. Differential scanning calorimetry (DSC) was performed using a DSC-822e/400 instrument, with an aluminum capsule as the holder and reference. Nitrogen was used as the purge gas, and the temperature was cycled between 25 and 200 °C, with a heating rate of 10 °C min^−1^.

### 2.4. Evaluation of the Effect of MTX and Its Derivatives on Cell Viability

#### 2.4.1. Cell Culture and Cells

The HeLa cell line (Sigma, St. Louis, MO, USA) was cultured in low-glucose Dulbecco’s Modified Eagle’s medium (DMEM), supplemented with 5% fetal bovine serum (FBS) and 100 U/mL penicillin; 100 μg/mL streptomycin was obtained from Invitrogen. The cell line was maintained in a 95% air and 5% CO_2_ atmosphere at 37 °C.

#### 2.4.2. Cell Viability Determination by MTS Assay

HeLa cells were exposed to experimental conditions in low-glucose DMEM supplemented with 5% FBS. Cells were exposed to MTX and β-CD/MTX for 1 h, then the culture medium was removed and cells were washed three times with phosphate-buffered saline (PBS). The cells were maintained in a 95% air, 5% CO_2_ atmosphere at 37 °C in the cell-culture medium for 72 h and their viability was determined using MTS assay (Promega, Madison, WI, USA). The MTX was dissolved with DMSO at a final concentration of 1% *v*/*v*. The untreated control cells were evaluated with and without 1% DMSO, to confirm that it did not affect HeLa cell viability. MTS, a tetrazolium compound, is bioreduced by cells to the formazan product. The absorbance of the formazan was determined with a colorimetric end-point *kit* according to the manufacturer’s instructions. A cell calibration curve was produced to ensure linearity during the study duration. The background was subtracted and data were expressed as percentages of surviving cells (mean ± SEM) relative to the control (cellular medium without DMSO).

#### 2.4.3. Effect of the Irradiated AuNPs + β-CD/MTX on Cell Viability

First, the AuNPs + β-CD/MTX system was irradiated with a 532-nm and 45-mW continuous laser for 15 min under sterile conditions. The HeLa cells were further incubated with an irradiated and nonirradiated (control) sample for 1 h to a final concentration of 0.1 mM, and cell viability was determined 71 h later (in triplicate) by MTS assay according to the manufacturer’s instructions.

#### 2.4.4. Data Analysis

All the results are presented as the mean and SEM, obtained from three or more independent experiments. Statistical analysis of the data was performed using GraphPad Prism 5 Software, Inc., (San Diego, CA, USA) ANOVA that was followed by the Bonferroni or Dunn’s post hoc tests, which were considered to be significant when *p* was less than 0.05.

## 3. Results and Discussion

### 3.1. Characterization of β-CD/MTX

**Figure 2:**
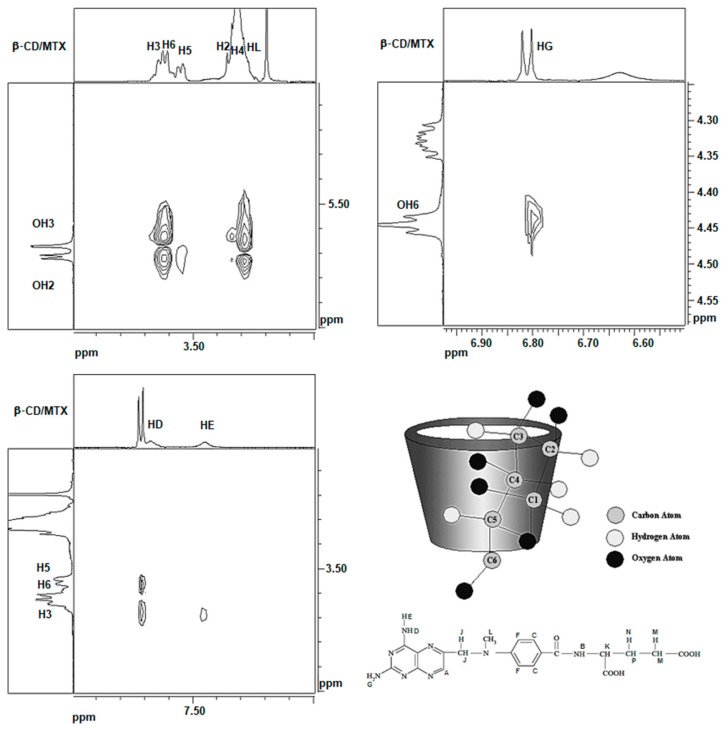
Segmented 2D-ROESY spectrum of β-CD/MTX in DMSO-d_6_ solvent and the schematic representation and proton assignment with numbers and letters for β-CD and MTX, respectively.

The 2D-ROESY NMR method was used to confirm the IC formation and to determine the intermolecular interactions of the guest (MTX) with the matrix (β-CD) during inclusion. The segmented 2D-ROESY spectrum, depicted in Figure 2 and Figure S3 (Supplementary Materials), illustrated the aforementioned interactions. The interactions of the external -OH_2_ and -OH_3_ protons of β-CD with the H_L_ of the drug were observed; additionally, the strong interaction between the H_3_ and H_5_ protons of the internal cavity of the matrix with H_D_, the amino proton of the pteridine ring of the MTX, and the weaker interaction of H_3_ with H_E_, another proton of the amino group near H_D_, that also belonged to MTX, were observed.

The interaction of the H_G_ proton of MTX with the group of external protons of the secondary hydroxyl group, OH_6_ of CD, was also observed. These protons are located in the narrowest rim of the cone, indicating that another functional amino group of the pteridine ring of MTX undergoes a dipolar interaction with the secondary hydroxyl of the matrix. These results indicate that the pteridine ring of MTX is located in the cavity of β-CD.

The absence of interactions between β-CD and the remainder of the MTX molecules confirms that p-aminobenzoic acid and glutamic acid waste were eliminated from the β-CD cavity. Therefore, it can be unequivocally confirmed that the IC presents a 1:1 matrix:guest stoichiometry.

The information obtained using the NMR spectroscopy is consistent with that obtained from a previous study that investigated the molecular modeling of the inclusion of MTX in the β-CD cavity [14].

### 3.2. Obtaining and Characterizing the Ternary System AuNPs+β-CD/MTX

**Figure 3:**
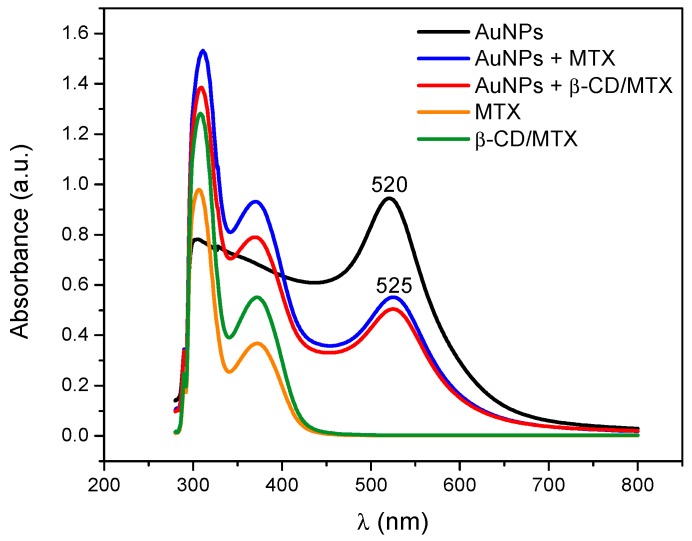
UV-Vis absorption spectra of MTX, inclusion compound (IC) and AuNPs stabilized with citrate ions, MTX molecules, and β-CD/MTX IC.

Figure 3 depicts the UV-Vis spectra of AuNPs that are stabilized with citrate, MTX, and β-CD/MTX IC. An absorption band at 520 nm corresponds to the characteristic surface plasmon band of spherical AuNPs with a diameter of 12 nm, stabilized with citrate ions [27]. When the citrate ions are exchanged with MTX or β-CD/MTX IC, a bathochromic shift of the plasmon band was observed from 520 to 525 nm due to the environmental changes around the gold nanoparticle. Furthermore, two bands were observed at 311 and 370 nm that corresponded with the MTX absorption bands, which were conjugated to the AuNPs [21].

**Figure 4:**
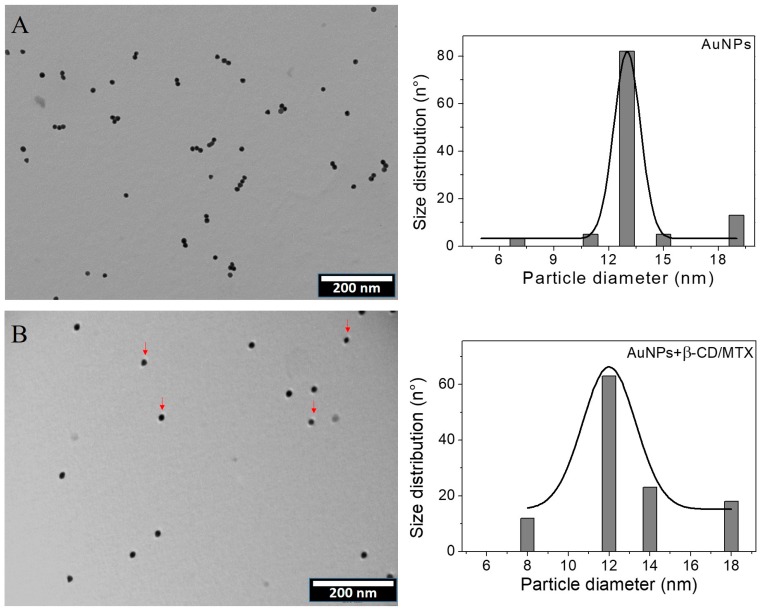
TEM micrographs and size histograms of AuNPs stabilized with citrate (**A**) and β-CD/MTX (**B**). The red arrows indicate the presence of an organic stained layer present around several gold spheres, which could correspond to the IC.

TEM provides information about the morphologies and sizes of the AuNPs and can, therefore, statistically determine the size of their population in different areas. Figure 4A depicts a TEM micrograph and histogram of AuNPs. A homogeneous distribution is observed with respect to both the shape and size. The histogram that is obtained from a population of 100 particles depicts an average diameter of 12 ± 2 nm, which is consistent with that exhibited by the 520 nm plasmon.

DLS indicated a hydrodynamic diameter of 19 nm with a polydispersity index (PDI) of 0.2. The value obtained by this technique was different from those obtained by TEM, which is because DLS provides information about the sizes of the particles that are present in the dispersion with their corresponding hydration sphere, i.e., the metal particle and its surroundings appear as citrate and associated ions.

Figure 4B and Figure S4 depict a TEM micrograph of the AuNPs that are coated with β-CD/MTX. Homogeneously distributed spherical particles of 12 ± 3 nm diameter were observed. The modification of the surface of the AuNPs with the IC maintains the shape and size unaltered. TEM micrograph shows an organic stained layer present around several gold spheres, which could correspond to β-CD/MTX. The histogram exhibits an average diameter of 12 ± 3 nm for a population of 100 particles. The hydrodynamic diameter obtained using DLS was 17.2 nm (PDI 0.2), which is consistent with the diameter obtained by TEM. The hydrodynamic diameter is reduced because the water molecules are reorganized due to the presence of the IC. It was reported that CD molecules exhibit a high capacity to bind water molecules via hydrogen bonds to the hydroxyl groups at the edges of their structures [23], decreasing the hydration sphere around the nanoparticle.

Qualitative analysis of the surface was performed (see Figure S5). The general XPS spectra between 0 and 1200 eV are observed to exhibit C, O, and N 1 s peaks. Peaks of 4p, 4d, and 4f Au orbitals are also observed. Further, we record the high-resolution spectra of the oxygen 1 s region in MTX without and after conjugation with the nanoparticles.

**Figure 5:**
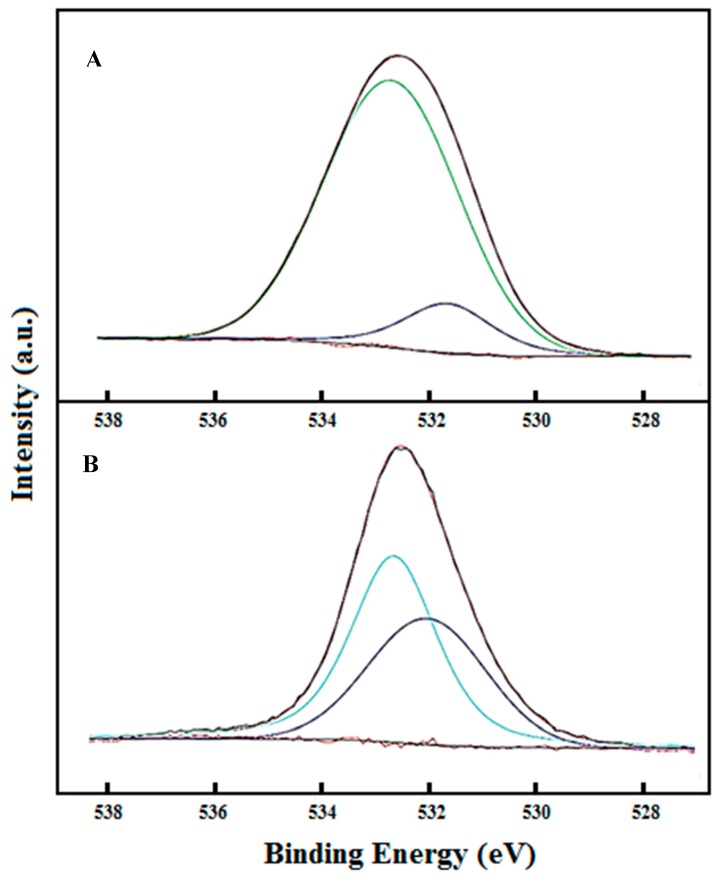
High-resolution XPS spectra of the O 1 s region of MTX in β-CD/MTX (**A**) and AuNPs + β-CD/MTX (**B**). The measured spectrum (black line) and the fitting curves (blue light and blue dark lines) are also depicted.

Figure 5A depicts the high-resolution spectra of oxygen 1s in the β-CD/MTX IC. Two bands were obtained by curve fitting at 531.43 and 532.50 eV. The MTX molecule contains two carboxylic acid groups with pKa values of 4.8 and 5.5. Both acids are deprotonated under working conditions at a pH value of 7.4. The band having a high intensity at 532.61 eV corresponds to the oxygen atoms of the carboxylate groups (–COO^−^). The band at 531.43 eV corresponds to the oxygen of the carbonyl group (>C=O) [17,28].

Figure 5B depicts a high-resolution spectrum of the IC that is conjugated to the AuNPs. Two bands are observed at 532.15 and 532.77 eV. The band at 532.15 eV corresponds to the oxygen atom of the carboxylate groups that interact with the AuNPs. The decrease in the binding energy of the 1s orbital electron from 532.61 to 532.15 eV is due to the increased shielding by AuNPs, which leads to increased electron density in the oxygen. Finally, the band at 532.77 eV corresponds to the carbonyl group. According to the studies that are related to the interaction of carboxylic acids with AuNPs, both the carboxylate groups of MTX are assumed to present weak interactions on the AuNP surfaces as compared with the strength of the covalent interactions [29].

**Figure 6:**
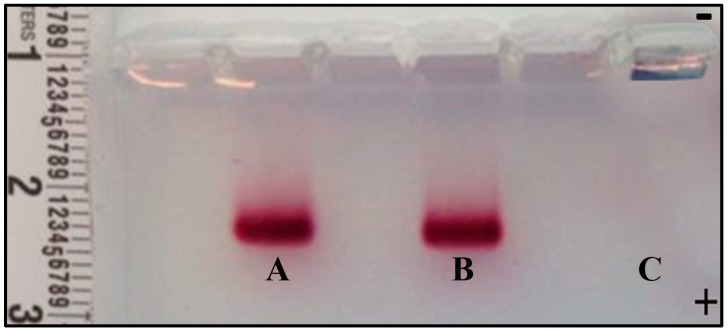
Electropherograms of AuNPs + β-CD/MTX (**A**), AuNPs + MTX (**B**), and AuNPs (**C**) on 1.0% agarose gel.

The mobility of supramolecular species was evaluated in the presence of an electric field using agarose gel electrophoresis. The migration of species was determined by their charge and particle size. Figure 6 depicts the agarose gel image and the respective migration of AuNPs + β-CD/MTX IC, AuNPs + MTX, and AuNPs species. AuNPs that are coated using β-CD/MTX (Sample A) exhibit less electrophoretic mobility to the anode compared to AuNPs that are coated using MTX (Sample B) (Figure 6). This is probably caused by the two negative charges on the MTX carboxylate ions that are partially masked by CD.

In the C lane, where the AuNPs were charged, a bluish coloration characteristic of colloid aggregation was observed. This is probably because the citrate ion fails to stabilize them under the observed working conditions (pH 9–10 due to the TAE buffer). This instability of AuNPs in buffer prevents migration, which is contrary to the expectations based on the size of the AuNPs and the triple negative charge afforded by the citrate ion.

The aforementioned results confirm that both samples (AuNPs + MTX and AuNPs + β-CD/MTX) exhibit a negative charge that induces their migration toward the anode. Furthermore, the AuNPs exhibited stability and integrity while being conjugated to the IC and while being coated by the MTX molecules under these working conditions.

**Figure 7:**
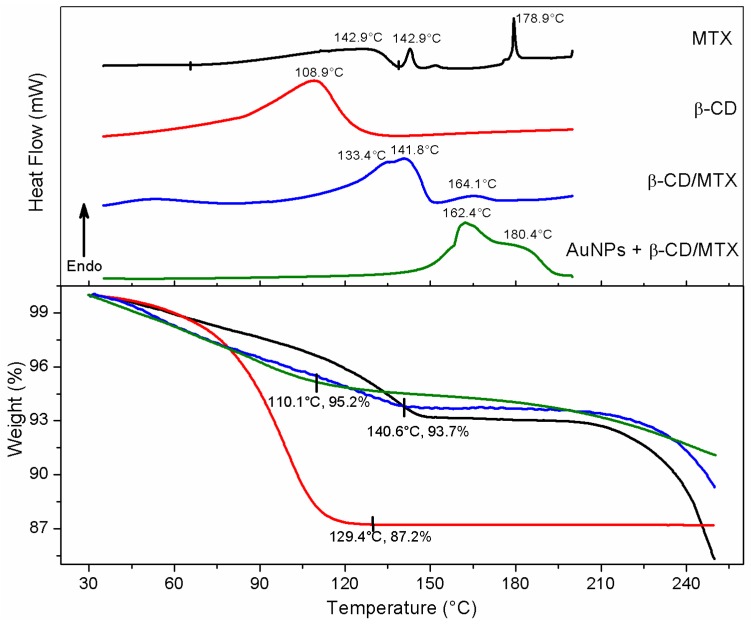
TGA and DSC analysis of MTX, IC, and AuNPs + IC.

TGA and DSC were performed to determine the thermal stability and to elucidate the thermal transitions of precursors of the IC and the ternary system. Figure 7 depicts the DSC analysis of MTX, and three endothermic peaks have been observed. The first broad peak, between 65.3 and 138 °C, corresponded to the thermal instability of the MTX trihydrate and therefore to the mass loss associated with these water molecules. The second endothermic peak, at 142°C, related to a possible pseudo melting or dissolution [30] and the third peak, at 178 °C, corresponded to its intrinsic melting point (174 °C) [15].

The DSC analysis of the β-CD matrix presented a single broad peak with a maximum at 109 °C. The thermal event was associated with a continuous mass loss from 30 °C to 129.4 °C, corresponding to a 12.8% mass of evaporated water molecules. After 130 °C, the matrix remained stable throughout the temperature range evaluated.

The β-CD/MTX IC thermic analysis showed a broad endothermic peak at 141.8 °C associated with an overlap of thermal events related to a loss of water molecules and pure MTX peak. The sample showed a continuous mass loss (6.3%) from 30 °C to 140.6 °C. The absence of free β-CD confirms the IC formation. The IC presented a possible amorphization of MTX with an increase in thermal stability [31]. The second peak at 164.2 °C was associated with a temperature-induced rearrangement of the IC. Similar results were observed for other ICs [22,23].

The DSC analysis of the IC conjugated with the AuNPs presents an endothermic peak at 163 °C with a shoulder at 180 °C. The mean peak was associated with a temperature-induced rearrangement of the IC. This is considered to be pharmacologically advantageous because the plasmonic heat generated by laser irradiation will produce irreversible leakage of the MTX guest from the β-CD cavity. The shoulder at 180 °C corresponds to the melting temperature of MTX which acquires a high stability inside the CD cavity [15]. The DSC of AuNPs + β-CD/MTX IC differs significantly from the IC. This difference may be explained based on the geometry of the inclusion. Proton-NMR studies depicted that only the pteridine ring is contained in the cavity of the β-CD molecule and that the remainder of the host structure, which interacts with the AuNPs, is excluded. Conjugation with the AuNPs occurs with the loss of electron density of the guest molecule, which favors the inclusion of the pteridine ring, providing increased stability to the system. It is also observed that there is no significant mass loss or decomposition by the sample between 30 °C and 250 °C. The sample decreases 8.93% which is attributable to the loss of water molecules [32].

### 3.3. Evaluation of the Effect of MTX and Its Derivatives on Cell Viability

**Figure 8:**
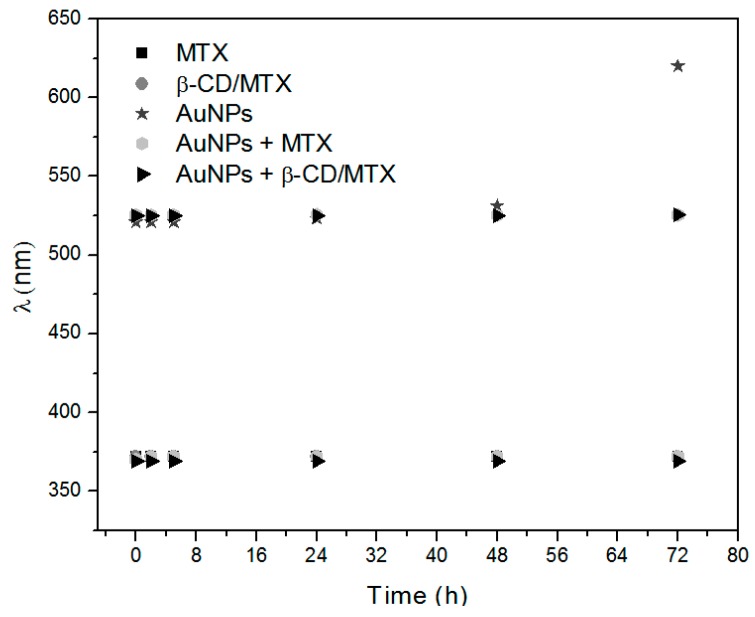
Schematic of the wavelengths of the characteristic absorption bands of MTX, IC, AuNPs + MTX, AuNPs + IC, and AuNPs at time zero and at 2, 5, 24, 48, and 72 h.

To confirm the stability of the MTX samples in a saline phosphate buffer (10 mM PBS), stability analysis was conducted by UV-Vis spectrophotometry. Figure 8 depicts a plot of the wavelengths of the characteristic absorption bands of MTX (370 nm) and AuNPs (520 nm) that are obtained from the absorption spectra of MTX, IC, AuNPs + MTX, AuNPs + β-CD/MTX, and AuNPs at different times (details Figure S6 in Supplementary Materials). These samples were stable in the PBS buffer, with the exception of AuNPs. This difference in the superficial plasmon band wavelength, which begins after 48 h and increases after 72 h, is introduced due to the colloidal suspension of AuNPs in the PBS buffer. However, when the AuNPs were conjugated with either MTX or IC, they remained stable during the evaluation time. Based on these results, a proliferation study was performed through MTS assay in the HeLa cells.

MTX is an analog of folic acid, binding to the folate receptors through its pteridine ring; this inhibits the action of the DHFR enzyme, which further interrupts the cell cycle [17,33,34]. To evaluate whether the inherent cytotoxicity of MTX was reduced when it was encapsulated by CD, the effects of MTX and IC on the viability of the HeLa cells at equivalent concentrations were compared.

**Figure 9:**
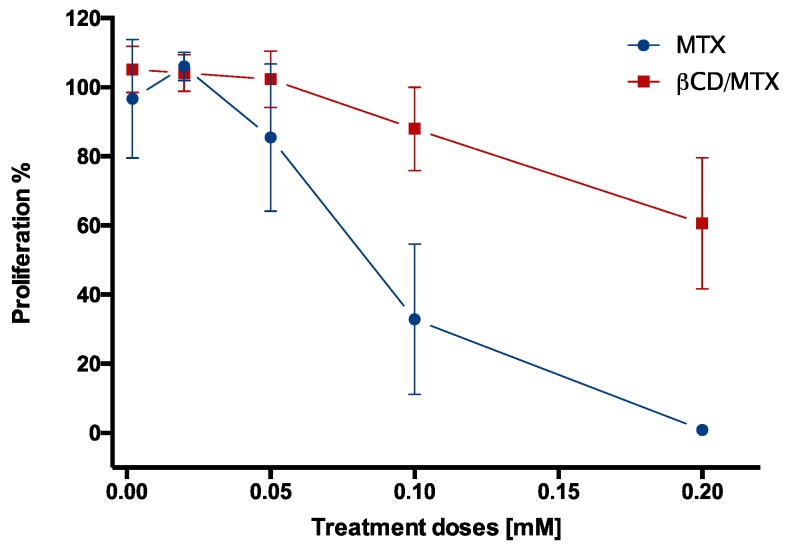
Comparison of cell viability of the HeLa cells treated with MTX or β-CD/MTX at different MTX concentrations (0.002–0.2 mM). The data are expressed as percentages of the live cells relative to the untreated cells (controls). The values represent the mean and SEM of three separate experiments in triplicate (* *p* < 0.05).

Figure 9 depicts a significant reduction in cell viability at high concentrations of free MTX (tested at 0.1 and 0.2 mM) with respect to β-CD/MTX IC. The 50% inhibitory concentrations (IC50) of MTX and the IC were calculated from the following equations obtained by nonlinear curve Fit (doseResp) in OriginPro software:

$$y=0.67+\frac{103.41-0.67}{1+10^{\left(0.08392-x\right)*-21.60524}}\;\mathrm{and}\;y=\frac{8.56}{1+e^{-2.62\left(x-0.20484\right)}},\;\mathrm{respectively}.$$

The IC50 obtained for MTX (0.0839 mM) was smaller than that obtained for β-CD/MTX IC (0.239 mM) because this required a concentration that was 2.8 times higher than that of MTX to significantly reduce cell viability. This protective effect of the IC is probably due to the inclusion of the pteridine ring of MTX inside the CD cavities, which prevents its interaction with the folate receptors and reduces its effect on cell proliferation. In order to evaluate the effects of β-CD/MTX IC and MTX on healthy cells, we used endothelial cells BEND3 and embryonic kidney cells HEK293T as a model brain. The treatment did not exert any effect on cell viability after 72 h of treatment, which is very relevant considering potential applications in terms of selectivity (Figure S7).

On the other hand, we tested the effects of AuNPs on cell viability by MTS assay. The nanoparticles did not affect cell viability in the assayed doses (Figure S8).

### 3.4. Evaluation of Cell Viability by MTS Assays of AuNPs + β-CD/MTX Samples with and without Irradiation

**Figure 10:**
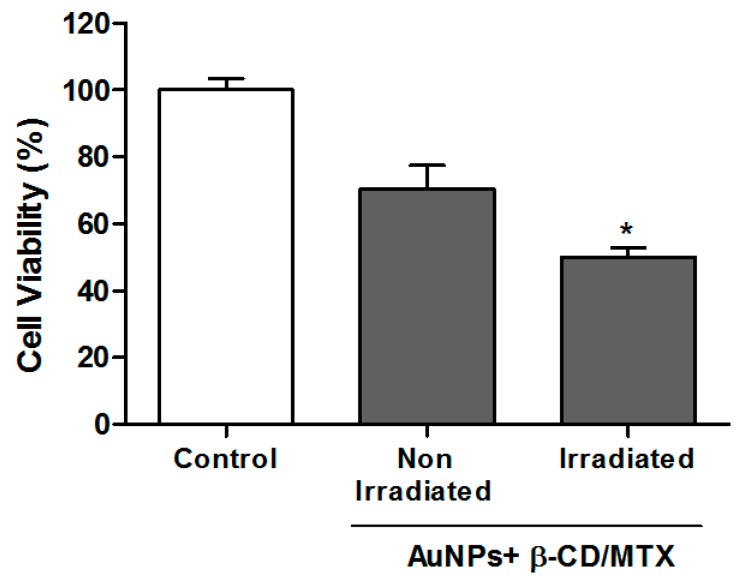
Test of the viability of the HeLa cells treated with AuNPs + β-CD/MTX, previously irradiated for 15 min with a 532-nm laser (50 mW). The HeLa cells were incubated for 1 h with AuNPs + β-CD/MTX (0.1 mM) (irradiated and nonirradiated) samples. After 71 h, cell viability was determined through MTS assay. The results are expressed as percentages compared with untreated cells and represent the mean and SEM of the three independent experiments in triplicate (* *p* < 0.05).

The AuNPs were conjugated with the IC to induce drug release from an intelligent delivery system using the photothermal effect of the AuNPs. To achieve this objective, an AuNPs + β-CD/MTX sample was irradiated with a continuous laser at 532 nm for 15 min. The HeLa cells were then incubated with these samples (irradiated and not irradiated) at a final concentration of 0.1 mM. We select this concentration because it significantly reduces cell viability (Figure 9) with MTX but not with β-CD/MTX IC. Figure 10 depicts a significant reduction in cell viability with respect to the nonirradiated control. This illustrates that the heat generated by the plasmonic effect of AuNPs is sufficient to disassemble the IC, which can cause the release of the MTX molecule. Moreover, we determined the release profile of MTX in the presence of irradiation following a protocol described in reference 24, observing that after 15 min of irradiation the 40 ± 9% of the free drug is released from the ternary system (See Figure S9A–C). Thus the free drug interacts with the folate receptors in cells, interferes with the nucleic acid synthesis, inhibits the cell proliferation and induces cytotoxic effects [17,33,34]. Furthermore, it has been widely established that MTX diminishes the antioxidant systems of cells [35,36,37]; these increased intracellular reactive oxygen species (ROS) levels develop a mitochondrial dysfunction and induce apoptosis [38,39]. This effect was not observed with the irradiated IC without nanoparticles (see Figure S10).

## 4. Conclusions

Purification and inclusion of MTX in the β-CD cavity were achieved, which also resulted in the formation of an IC with 1:1 stoichiometry. An MTS viability assay on the HeLa cell line treated using MTX and β-CD/MTX IC depicted that the inclusion of the drug in CD reduced the cytotoxic effect as compared to that observed in free MTX.

An MTX nanocarrier system was designed to ensure controlled drug release using the photothermal effect of AuNPs. Thus, the β-CD/MTX samples were conjugated to AuNPs and were irradiated using a 532-nm laser (laser of wavelength tunable with the plasmon), which caused a decrease in cell viability relative to that observed in the case of nonirradiated control after the release of MTX from the β-CD cavities.

These results may contribute to the treatment of cancer and promote the design of a drug delivery strategy to control the local and temporary photothermal release of antitumor drugs.

## Supplementary Materials

The following are available online at http://www.mdpi.com/2079-4991/8/12/985/s1. Figure S1. ^1^H-NMR spectra of reported mannitol, MTX + mannitol, purified MTX and reported MTX, Figure S2. Schematic representation of the methodologic protocol for the synthesis of colloidal AuNPs and their conjugation with the β-CD/MTX, Figure S3. 2D-ROESY spectrum of β-CD/MTX IC in DMSO-d_6_ solvent, Figure S4. TEM micrograph of AuNPs stabilized with β-CD/MTX IC. The red arrows indicate the presence of an organic stained layer present around several gold spheres, which could correspond to the IC, Figure S5. XPS general spectrum of AuNPs+β-CD/MTX IC, Figure S6. Absorption spectra of (a) MTX, (b) IC, (c) AuNPs + MTX, (d) AuNPs + IC and e) AuNPs, in PBS at time zero, 2, 5, 24, 48 and 72 h, Figure S7. Viability after applying treatments of 0.1mM β-CD/MTX. Viability evaluated using the MTS assay over (A) BEND3 cells and (B) HEK293 for 24 h (*n* = 3), Figure S8. Effects of AuNPs on cell viability of HeLa cells. Viability was evaluated using the MTS assay over HeLa cells for 24 h (*n* = 3), Figure S9A. Absorbance spectra of MTX in chloroform at different concentrations. The insert shows the calibration curve obtained measuring the absorbance intensity at 266 nm vs the concentration of MTX. (*n* = 2), Figure S9B. Release percentage of MTX under irradiation (left) or without irradiation (right) for different cumulative times (*n* = 3). The irradiation was performed with a laser of 532 nm (50 mW), Figure S9C. Absorbance spectra of organic phase different accumulative times with and without irradiation with a 532nm laser (50 mW). (*n* = 3), Figure S10. Test of HeLa cells viability treated with β-CD/MTX, irradiated previously during 15 min with a 532-nm laser (45 mW). Then HeLa cells were incubated for 1 h with both β-CD/MTX (0.1mM) (irradiated and non-irradiated) samples. After 71 h it was determined, the cell viability through MTS assays. The results are expressed as percentages compared with untreated cells, and represent the mean and SEM of three independent experiments in triplicate (* p < 0.05). Table S1. Chemical shifts of MTX + mannitol, purified MTX and reported MTX. Schematic representation and proton assignment of MTX.
