# Supplementary material for: Photothermally Controlled Methotrexate Release System Using β-Cyclodextrin and Gold Nanoparticles
Source: Nanomaterials (Basel). 2018 Nov 28;8(12):985. doi: 10.3390/nano8120985 (PMC6315352; doi:10.3390/nano8120985)
Supplement: Supplementary file 1 [file nanomaterials-08-00985-s001.zip › nanomaterials-374614-SI.pdf]

Supplementary Material

Photothermally Controlled Methotrexate Release System Using β-Cyclodextrin and Gold Nanoparticles

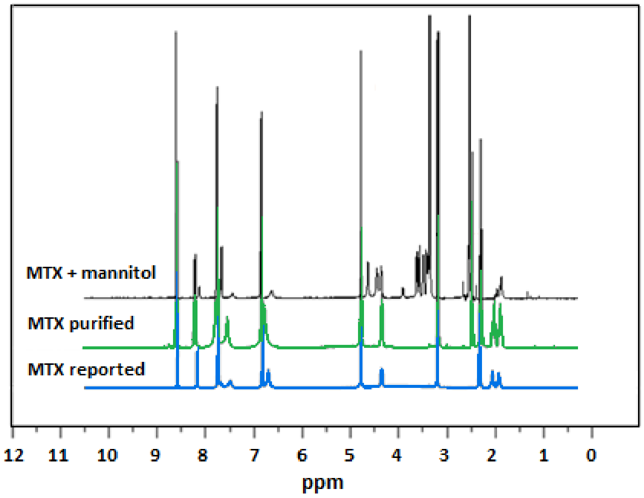

Figure S1. ^{1}H-NMR spectra of reported mannitol, MTX + mannitol, purified MTX and reported MTX.

Table S1. Chemical shifts of MTX + mannitol, purified MTX and reported MTX. Schematic representation and proton assignment of MTX.

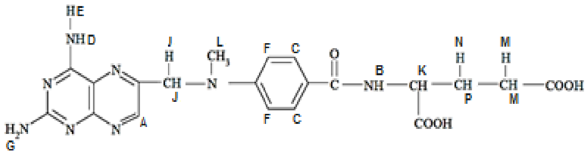

Proton	MTX+Manitol (ppm)	MTX Purified (ppm)	MTX Reported (ppm)
A	8.57	8.46	8.59
B	8.09	8.24	8.20
C	7.63	7.76	7.74
D	7.62	7.73	7.70
E	7.39	7.54	7.50
F	6.82	6.82	6.83
G	6.59	6.76	6.70
J	4.32	4.35	4.37
K	3.16	3.24	3.21
L	1.00	1.05	2.03
M	2.35	2.32	2.33
N	1.93	2.07	2.07
P	1.83	1.92	1.94

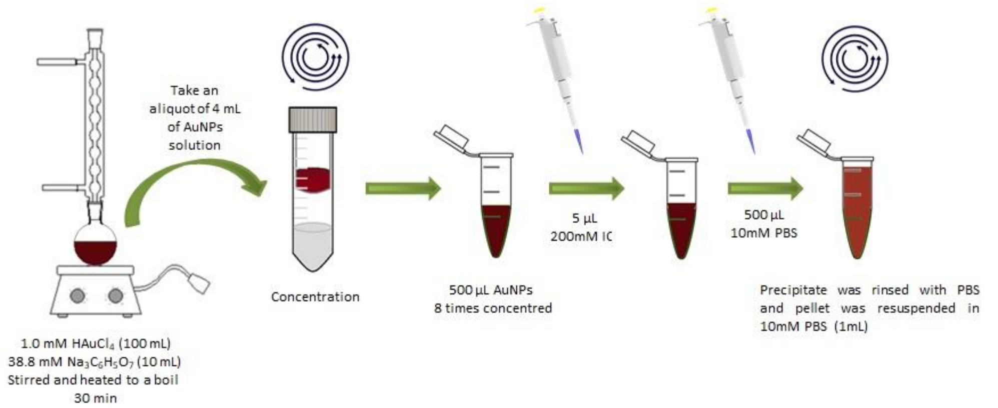

Figure S2. Schematic representation of the methodologic protocol for the synthesis of colloidal AuNPs and their conjugation with the β-CD/MTX.

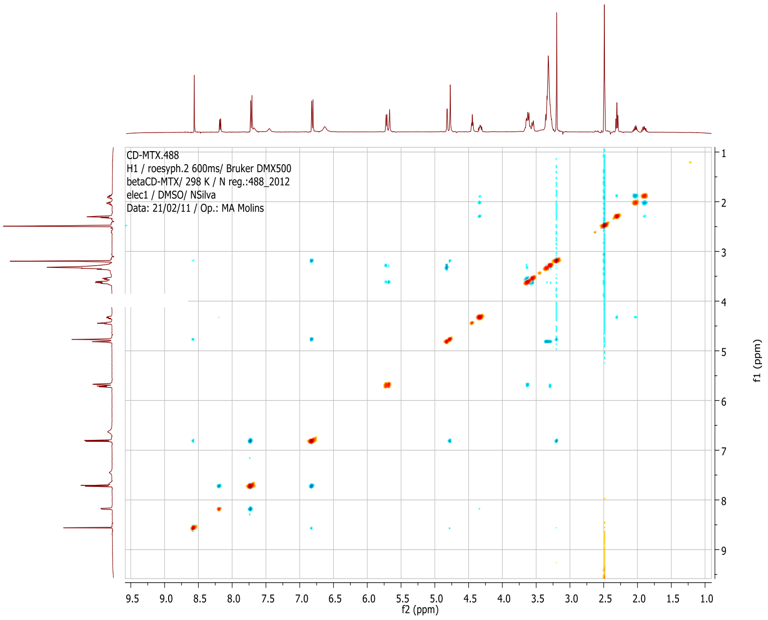

Figure S3. 2D-ROESY spectrum of β-CD/MTX IC in DMSO-d_6 solvent.

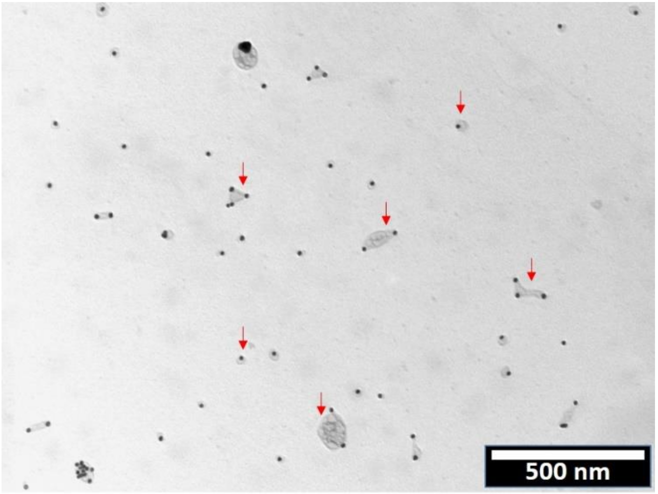

Figure S4. TEM micrograph of AuNPs stabilized with β-CD/MTX IC. The red arrows indicate the presence of an organic stained layer present around several gold spheres, which could correspond to the IC.

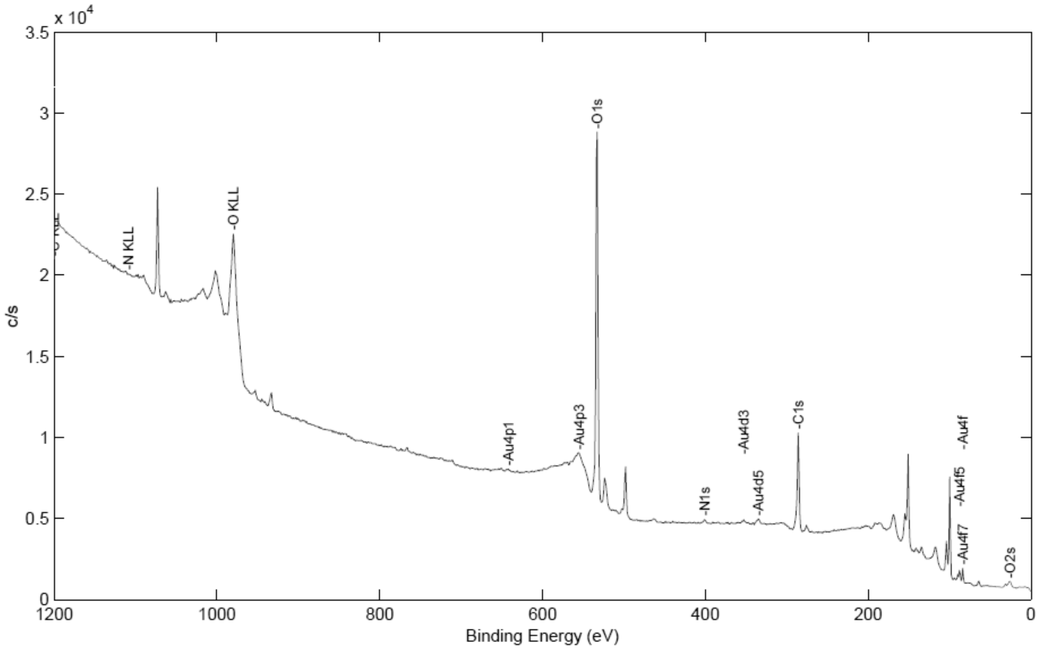

Figure S5. XPS general spectrum of AuNPs+β-CD/MTX IC.

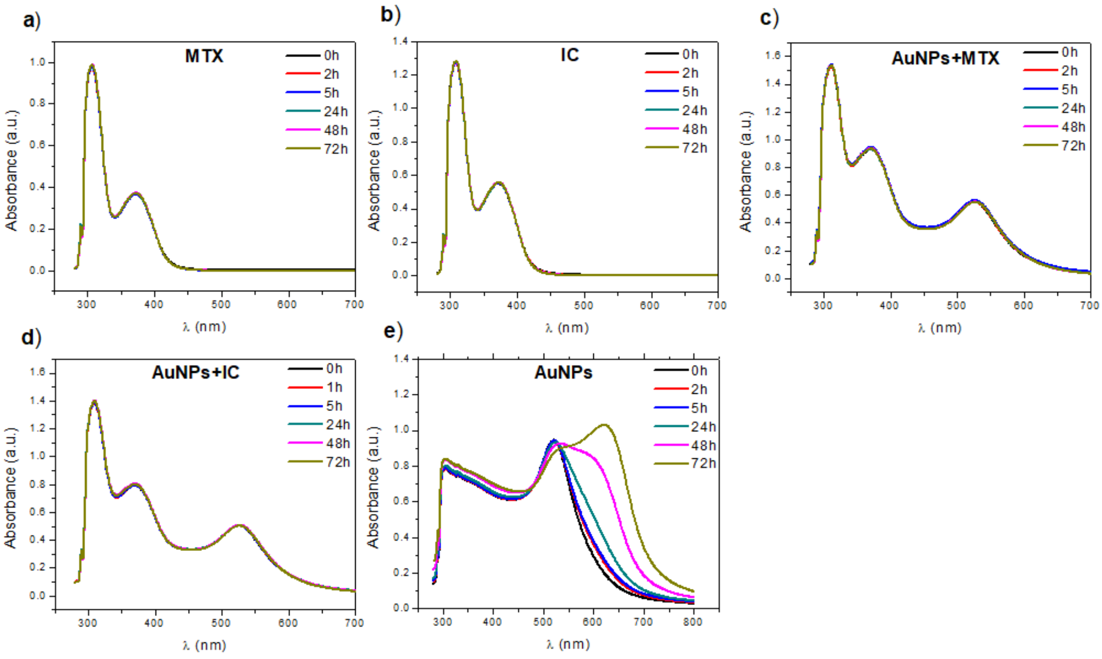

Figure S6. Absorption spectra of (**a**) MTX, (**b**) IC, (**c**) AuNPs + MTX, (**d**) AuNPs + IC and e) AuNPs, in PBS at time zero, 2, 5, 24, 48 and 72 h.

Cell viability assays

Cell culture conditions

Human epithelial adenocarcinoma (HeLA, ATCC) and mouse endotelial (BEND-3, ATCC) cells lines were cultured in DMEM High Glucose (GIBCO) supplemented with 10% FBS (Biological Industries) and antibiotics (100 U/mL penicillin and 100 μg/mL streptomycin, GIBCO) [1]. Human embryonic kidney cells (HEK293T, ATCC), cell line was cultured in RPMI 1640 medium (GIBCO) containing 10% FBS (Biological Industries) and antibiotics (100 U/mL penicillin and 100 μg/mL streptomycin, GIBCO) [2]. Cells were cultured at 37°C and 5% CO_2.

Viability assays

Cells were seeded in 96-well plates at a density of 3 × 10^4 cells per well and incubated overnight in culture medium. Then, the medium was replaced with fresh culture medium containing 10% of different treatments (AuNP; CI-MTX, MTX, CI-MTX-AuNP) and maintained by 1 h in these conditions. Later, the medium was replaced with fresh culture medium to maintain cells by 72 h. Finally, cell proliferation was evaluated replacing medium with fresh culture medium containing 10% of MTS® assay (CellTiter 96® Aqueous Non-Radioactive Cell Proliferation Assay; Promega, Madison, WI). The colorimetric product is detected as absorbance at 490 nm on a Multiscan Reader (Synergy-H4, Biotek) [3]. Background values contributed by excess cell debris and bubbles obtained by measuring at 650 nm were subtracted.

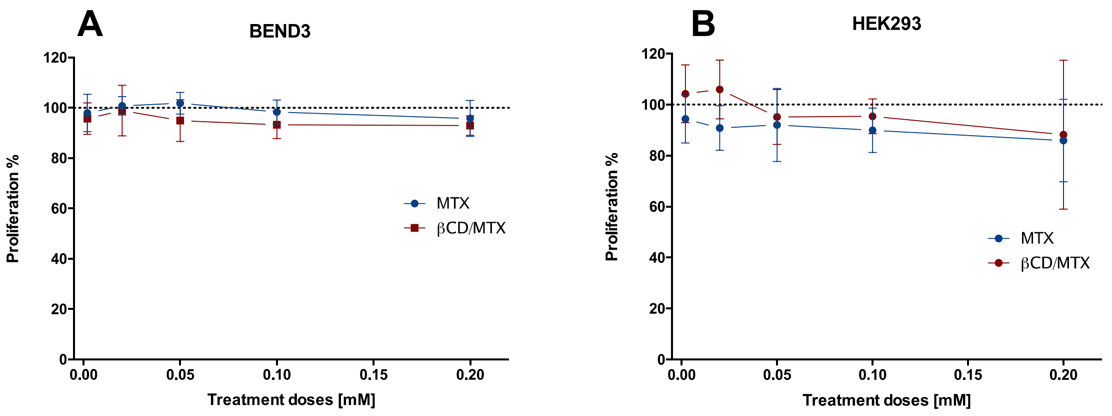

Figure S7. Viability after applying treatments of 0.1mM β-CD/MTX. Viability evaluated using the MTS assay over (**A**) BEND3 cells and (**B)** HEK293 for 24 h (n=3).

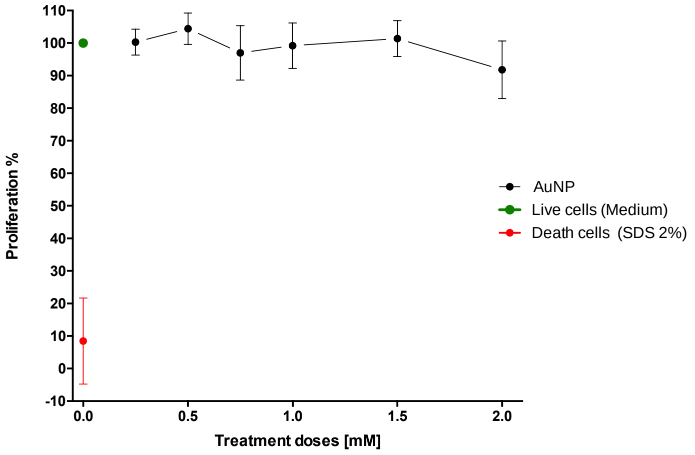

Figure S8. Effects of AuNPs on cell viability of HeLa cells. Viability was evaluated using the MTS assay over HeLa cells for 24 h (n=3).

Evaluation of the release profile of MTX from the ternary system (AuNPs + β-CD/MTX)

Spectrophotometric experiments have been performed to evaluate the MTX release percentage from the ternary system as function of irradiation time according with reference 24. Free MTX is soluble in chloroform unlike the IC. A two phase system with an aqueous phase that contain the ternary system (AuNPs + β-CD/MTX) and an organic phase of chloroform were performed. The aqueous phase was irradiated using a continuous laser of 532 nm (50 mW). The photothermal effect of the AuNPs irradiated cause the disassemble of the IC and the release of MTX molecules toward the organic phase being able to be quantified by an absorption band at 266 nm in the organic phase.

Firstly, a calibration curve has been performed for different concentrations of the MTX in clorophorm (Figure S9A). Secondly, the aqueous phase in the quartz cuvette with the two phase system was irradiated for different times and the absorbance spectra of the organic phase were determined (Figure S9B,C). The time course for the disassemble of the complex related with the MTX release was obtained. After 15 min of laser irradiation a 40±9.1% of MTX was released (Figure S9B,C). The ternary system without irradiaton shows release of the MTX, but insignificant with respect to the photothermically activated system (Figure S9B,C).

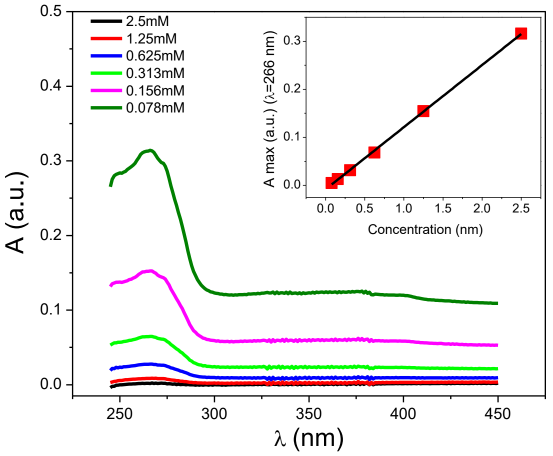

Figure S9A. Absorbance spectra of MTX in chloroform at different concentrations. The insert shows the calibration curve obtained measuring the absorbance intensity at 266 nm vs the concentration of MTX. (*n*=2).

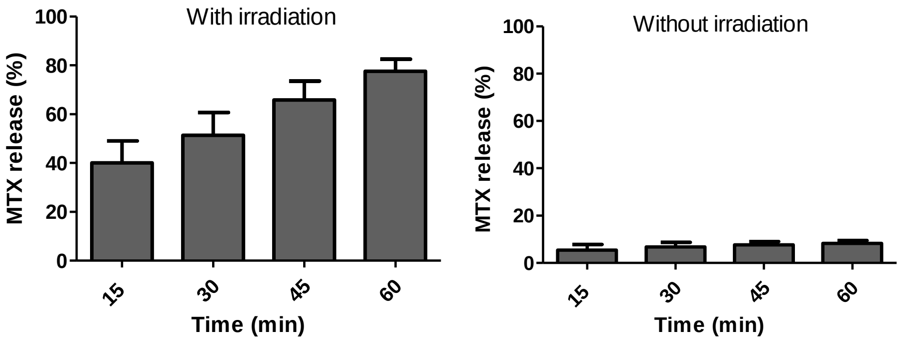

Figure S9B. Release percentage of MTX under irradiation (left) or without irradiation (right) for different cumulative times (*n* = 3). The irradiation was performed with a laser of 532 nm (50 mW).

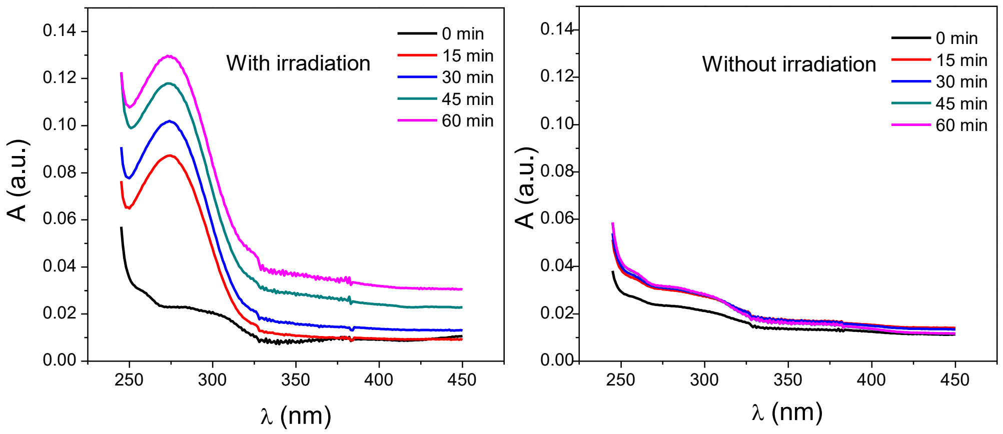

Figure S9C. Absorbance spectra of organic phase different accumulative times with and without irradiation with a 532 nm laser (50 mW). (*n=3*).

Methodology:

The release profile has been determined according with reference 24. A calibration curve was obtained by measuring the absorbance of different concentrations of MTX in chloroform. The absorbance spectra were taken in a Shimadzu UV-3101PC spectrophotometer using a quartz cuvette of 1mL. The absorbance intensity was determined at 266 nm obtaining the following equation ($y = 0.12885x - 0.00257$) A two-phase system was formed for irradiation experiments. In a quartz cuvette chloroform (300 μL) and 1 mM of MTX in the ternary system solution (200 μL) were added. The upper phase of the solution that contained the AuNPs + β-CD/MTX sample was irradiated with a 532 nm and 50-mW continuous laser during different cumulative times (15, 30 and 45 min). Then, the absorbance of MTX was measured in the lower phase of chloroform, without separating the two phases. The absorbance intensity at 266 nm was related with the MTX concentration and finally with the release kinetics as a function on irradiation time was obtained. A solution of the ternary system without irradiation was evaluated as control.

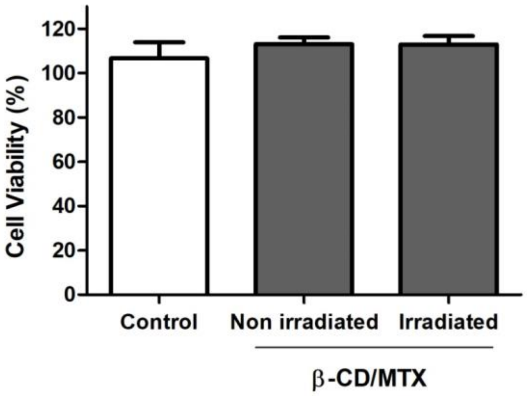

Figure S10. Test of HeLa cells viability treated with β-CD/MTX, irradiated previously during 15 min with a 532-nm laser (45 mW). Then HeLa cells were incubated for 1 h with both β-CD/MTX (0.1mM) (irradiated and non-irradiated) samples. After 71 h it was determined, the cell viability through MTS assays. The results are expressed as percentages compared with untreated cells, and represent the mean and SEM of three independent experiments in triplicate (* $p < 0.05$).

References

1. Urra, H.; Torres, V.A.; Ortiz, R.J.; Lobos, L.; Diaz, M.I.; Diaz, N.; Hartel, S.; Leyton, L.; Quest, A.F. Caveolin-1-enhanced motility and focal adhesion turnover require tyrosine-14 but not accumulation to the rear in metastatic cancer cells. *PLoS One* **2012**, *7*, e33085.
2. Torres, V.A.; Tapia, J.C.; Rodriguez, D.A.; Lladser, A.; Arredondo, C.; Leyton, L.; Quest, A.F. E-Cadherin Is Required for Caveolin-1-Mediated Down-Regulation of the Inhibitor of Apoptosis Protein Survivin via Reduced β-Catenin-Tcf/Lef-Dependent Transcription. *Mol Cell Biol*, **2007**, *27*, 7703–7717.
3. Adura, C.; Guerrero, S.; Salas, E.; Medel, L.; Riveros, A.; Mena, J.; Arbiol, J.; Albericio, F.; Giralt, E.; Kogan, M.J. Stable Conjugates of Peptides with Gold Nanorods for Biomedical Applications with Reduced Effects on Cell Viability. *ACS Appl. Mater. Interfaces*, **2013**, *5*, 4076–4085.
